# Genome‐wide identification of endogenous retrovirus elements and their active transcription in mink genome

**DOI:** 10.1002/mlf2.12074

**Published:** 2023-06-30

**Authors:** Zheng Li, Qing Wang, Na Lv, Guojin Xu, Xuemei Yang, Baoli Zhu

**Affiliations:** ^1^ CAS Key Laboratory of Pathogen Microbiology and Immunology Institute of Microbiology, Chinese Academy of Sciences Beijing China; ^2^ University of Chinese Academy of Sciences Beijing China; ^3^ Jiangxi Science and Technology Normal University Nanchang China; ^4^ Jinan Microecological Biomedicine Shandong Laboratory Jinan China; ^5^ Beijing Pediatric Research Institute Beijing China; ^6^ Department of Pathogenic Biology, School of Basic Medical Sciences Southwest Medical University Luzhou China

**Keywords:** endogenous retrovirus, mink, tissue‐specific, transcriptome

## Abstract

Mammalian endogenous retroviruses (ERVs) are ancient retroviruses that have been integrated into genomes. ERVs were believed to be inactive until the discovery of ERV transcription in the mouse genome. However, the transcription level and function of ERV elements in mammalian genomes are not well understood. In this study, we performed the first genome‐wide scanning of ERV loci in the American mink (*Neogale vison*) genome (NeoERV) followed by transcriptomic analysis to detect actively transcribed NeoERV elements. A total of 365,791 NeoERV loci were identified, and161,205 (44%) of these loci were found to be actively transcribed based on transcriptomic data from three types of tissues (amygdala, trachea and lung). More than one third of the actively transcribed NeoERV loci were tissue‐specific. Furthermore, some of the active loci were associated with host gene transcription, and the level of NeoERV transcription was positively correlated with that of host genes, specifically when active loci were located in overlapped gene regions. An in‐depth analysis of the envelope protein coding *env* gene showed that, in general, its transcription level was higher than that of NeoERVs, which is believed to be associated with host immunity.

## Impact statement

Mink is an important economic animal that has also been used as an animal model to investigate the virus infection process that may be influenced by the endogenous retrovirus (ERV) elements integrated in the mink genome. We performed genome‐wide scanning of ERV elements in the mink genome and identified active loci of mink ERV elements in three different types of tissues. We found that the distribution and transcription levels of the active loci were tissue specific. The results will be helpful to investigations of the immunity of mink when it is used as an animal model.

## INTRODUCTION

During millions of years of evolution, mammalian host genomes accumulated a variety of endogenous retroviruses (ERVs) that were integrated and remained inactive. The ERV elements in mammlian genomes can be highly diverse and have been classified into seven genera^1^. The majority of ERV sequences in the genomes are incomplete and dispersed, which can be defined as “solo” and “slimmed down,” respectively, and a small portion of them can be defined as “complete” ERVs that is consisted of three genes, the *gag*, *pol*, and *env* flanked by two long terminal repeats (LTRs). The *gag* gene encodes a structural matrix and a capsid protein, the *pol* gene encodes a reverse transcriptase and an integrase, and the *env* gene encodes a glycoprotein that determines host cell tropism^2^. In human, there is no evidence of active human endogenous retroviruses (HERVs) that can undergo transmission, mainly because of the numerous mutations (e.g., insertions and substitutions) acquired in the course of evolution^3^. The “slimmed down” ERVs are generally found to be truncated by insertions, deletions, and substitutions, causing the loss of partial or whole ERV open reading frames and always lacking one or more coding sequences. The “solo” ERVs contain only two LTRs, most commonly generated by the recombination of two LTRs in general^4^, and represent the majority of the ancient ERVs found in mammals.

Although most ancient ERVs are incomplete, some of them were found to produce partial functional proteins or noncoding RNAs in mouse and human. The expression of *env* genes in “slimmed down” or “complete” ERVs may be crucial for normal host physiological processes and for maintaining the homeostasis of host immunity^5^. However, the abnormal expression of such ERV elements and LTRs can disturb the balance of host physiology and immunity, and lead to different types of diseases^6^. The best‐known example of this is syncytin‐1, a protein coded by *env* that regulates placental morphogenesis in human, mouse, and other placental animals^5^. When abnormally expressed, syncytin‐1 can act as an inflammatory factor in astrocytes and microglial cells, causing oligodendrocyte death and demyelination^7^, which can lead to multiple sclerosis (MS) disease. Expression of the *env* gene of human HERV‐W, a retroviral element associated with multiple sclerosis, produces an envlope pretein that can induce abnormal cytokine scretion that contributes to the inflammatory process in multiple sclerosis^8^. Abnormal expression of human HERV‐K has also been associated with cancer development because HERV‐K encodes the accessory proteins Rec and Np9 that have carcinogenic functions, particularly in germline cell cancer^9^. Together, these findings confirm that the expression of ERV elements is common in all mammalian genomes, and that their abnormal expression may lead to the development of diseases in the host.

Mink is a small mammal that has been used as an animal model for human disease studies. For example, mink can be infected by SARS‐CoV‐2 virus in a manner similar to that of human^10^, and therefore could be a potential animal model for studies of severe and fatal COVID‐19^11^. Pathogen infections have been linked previously with ERVs and the expression of ERV elements^12^. The American mink (*Neogale vison*) draft genome has a total length of 2564 MB and 4.1% of the genome contains ERV elements^13^. However, no systematic research aimed at identifying NeoERVs in the mink genome has been reported so far. In this study, we performed the genome‐wide identification of NeoERV elements using RepeatMasker software to identify all possible NeoERV loci in the mink genome. We also used the transcriptomic data to identify the transcripts at NeoERV loci. The NeoERV loci that produced transcripts were called active loci, and NeoERV loci that did not produce transcripts were called inactive loci. Our study provides the first overview of the genome‐wide distribution of NeoERV loci as well as transcriptomic data for the active loci in the mink genome.

## RESULTS

### NeoERVs make up 4.1% of the whole mink genome

We identified 365,791 NeoERV sequences in the mink draft genome^13^ using RepeatMasker. We divided the identified NeoERV sequences into four classes, ERV1, ERVK, ERVL, and ERVL‐MaLR, using the Repbase database^29^, which is used to classify all mammalian ERVs (Table 1). The NeoERV sequences made up 4.1% of the whole genome, which is lower than that of HERVs (8.7%) and mouse ERVs (11.5%) and comparable to that of cow ERVs (3.2%). The ERVL‐MaLR elements were the largest proportion (46.8%) of the NeoERV elements, which is comparable to that of the HERV elements (46.7%). The ERVL, ERV1, and ERVK elements accounted for percentages of 29.9%, 15.1%, and 8.2% of the NeoERV elements, respectively. Among the HERV elements, the ERVK elements were only 1.5%. The distribution of NeoERV elements varied on different chromosomes. Chromosome 1 had the highest density (12.5%) of NeoERV elements, and the largest proportion of them were ERVL‐MaLR elements. Most of the NeoERV elements (71.4%) were located in intergenic regions. Approximately 28.5% of the NeoERV elements were found in intragenic regions, and 98.9% of them were in intronic regions.

**Table 1:** Distribution of NeoERV elements in the mink genome.

	ERV1					ERVK				
	Intragenic					Intragenic				
Chr	Intron	Exon	Others	Intergenic	Overlapped region	Intron	Exon	Others	Intergenic	Overlapped region
1	1774	12	18	4930	10	1197	11	3	2414	2
2	1079	4	7	3539	10	879	13	1	1541	2
3	1121	2	17	3521	5	978	4	6	1606	3
4	1042	8	5	3583	3	831	5	4	1713	5
5	674	3	5	2480	3	561	2	1	1186	3
6	1274	6	16	3538	12	992	6	2	1611	8
7	1022	6	7	3279	12	720	6	4	1470	3
8	735	5	2	1908	4	578	8	2	1112	1
9	459	2	6	1319	3	401	4	1	638	1
10	477	2	6	1053	2	287	3	1	427	0
11	1327	3	13	3811	6	929	8	5	2041	3
12	976	5	5	2129	7	611	12	1	1005	0
13	798	3	10	2130	4	562	8	2	1053	3
14	290	5	3	594	1	271	1	0	209	0
X	889	9	6	2784	6	539	7	2	1434	4

	ERVL					ERVL‐MaLR				
	Intragenic					Intragenic				
Chr	Intron	Exon	Others	Intergenic	Overlapped region	Intron	Exon	Others	Intergenic	Overlapped region
1	3492	10	15	10,442	26	6062	20	20	14,988	24
2	2244	11	14	5939	20	4047	25	25	9485	33
3	2385	13	12	6639	14	4296	17	17	10,520	18
4	2332	7	9	6606	10	3930	20	20	9482	14
5	1561	5	15	4625	7	2931	17	17	6986	11
6	2866	11	19	7960	12	5251	14	14	11,473	22
7	2127	17	15	5401	19	3897	35	35	8106	22
8	1637	2	11	4799	9	3035	14	14	8153	22
9	1005	2	12	2822	5	1880	4	4	4345	10
10	686	7	15	1861	3	1287	9	9	2737	5
11	2465	7	23	8912	18	4070	22	22	11,482	11
12	1609	3	10	4180	1	3082	9	9	6643	21
13	1757	3	12	4701	7	2969	13	13	6607	7
14	657	5	8	1035	4	1628	15	15	1993	12
X	1103	5	18	4304	11	1815	25	25	5974	14

ERV, endogenous retrovirus.

### Core and tissue‐specific active loci of NeoERV elements in three tissues

**Figure 1:**
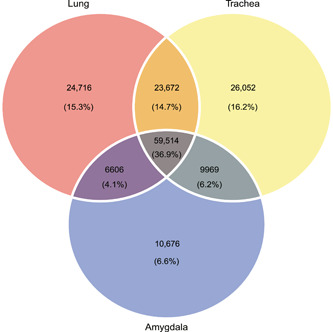
Expression of active loci of NeoERV elements in three types of mink tissues. Venn plot of the expression of active loci of NeoERVs in three tissues is shown. Red, yellow, and blue circles indicate the active loci in lung, trachea, and amygdala, respectively. The part of the circles that does not overlap with the other two circles indicates the active loci of NeoERVs that occurred in only one type of tissue. The parts of two circles that overlap indicate the active loci of NeoERVs that occurred in two tissues. The parts of three circles that overlap indicate core active loci that occurred in all tissues.

The genome‐wide transcription status of ERV elements in the mammalian genomes is still largely unknown, except for HERVs, where the expression of 3220 loci of proviral HERVs has been determined. However, these proviral HERV elements make up only approximately 1% of all HERV sequences^15^, and therefore do not provide a whole‐genome view of HERV expression. We used the RepeatMasker annotation tools to determine the total number of NeoERVs and their genome locations (gene loci), and used whole‐genome transcriptomic data to determine the active loci of the NeoERV elements. We identified 365,791 NeoERV elements in the whole genome of three mink tissues (amygdala, trachea, and lung). Using the whole genome transcription data, we identified 161,205 active NeoERV elements that generated specific transcripts, defined as “active loci” (Figure 1), and 204,586 NeoERV elements that had no transcripts, defined as “inactive loci” (Table S1). We identified 59,514 active loci (36.9% of the total active loci) that were common in all three tissues and named them core active loci, as well as 119,207 active loci in trachea, 114,508 in lung, and 86,765 in amygdala. We compared the active loci among the three tissues and found 61,444 active loci that were tissue‐specific (26,052 for the trachea, 24,716 for the lung, and 10,676 for the amygdala), accounting for 38.1% of the total active loci in all three tissues. Trachea had the largest number of tissue‐specific active loci, followed by lung and amygdala, which had a much lower number of active loci as shown in Figure 1. Most of active loci of the NeoERVs were in intergenic regions, 57.3% (65,654/114,508) for lung, 58.9% (70,304/119,207) for trachea, and 54.8% (47,604/86,765) for amygdala tissues (Table S1). Together, these results showed that most of NeoERV loci (80%) as well as active loci (60%) were located in intergenic regions, and approximately one third of the total NeoERV elements were active with true transcripts.

### Transcription levels of core active loci of NeoERV elements

**Figure 2:**
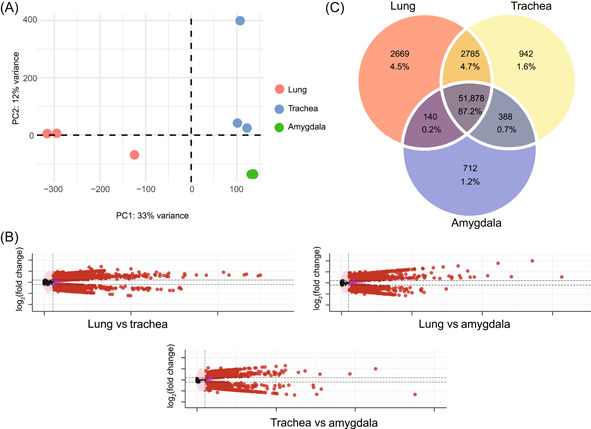
Expression levels of active loci of NeoERV in three types of mink tissues. (A) Relationships of different types of mink tissues by principal component analysis. (B) Volcano plot showing differential ERV expressions between different three mink tissues. The differentially expressed NeoERVs are highlighted in red. (C) A Venn plot of the differential expression of the active NeoERV loci in three types of tissues. The part of the circles that does not overlap with the other two circles indicates the active loci of NeoERVs that were upregulated in only one type of tissue. The parts of two circles that overlap indicate the active loci of NeoERVs were upregulated in two tissues. The parts of three circles that overlap indicate core active loci that were not tissue‐specifically expressed.

Although host genes are known to show tissue‐specific expression, the transcription patterns of NeoERV elements in different tissues are largely unknown. We found that more than one third of the active loci of NeoERVs were tissue‐specific, and we assumed that the transcription levels of the active loci may be different in different types of tissues. To quantify the transcription levels of the active loci of NeoERVs, we calculated the transcript per million (TPM) values of active loci in the three tissues. We conducted a principal component analysis (PCA) based on TPM values that were calculated from the retro‐transcriptomic data obtained from mink lung, trachea, and amygdala tissues (Figure 2A). The transcription levels of all the active loci of NeoERVs among the three tissues (lung, trachea, and amygdala) were compared using DESeq2^16^ (Figure 2B). The results showed that the transcription levels of 2669, 942, and 712 core active loci were upregulated in the lung, trachea and amygdala tissues, respectively (Figure 2C). In the amygdala tissue, 4192 active loci were downregulation, which is approximately six times higher than the number of active loci that were upregulated (712), implying that the transcription of active loci of NeoERVs in the amygdala may be associated with the immune status of healthy individuals. Therefore, a set of 4323 active loci of NeoERV elements that were tissue‐specifically transcribed were identified in the three tissues, in addition to the tissue‐specific active NeoERV loci (61,444) (Figure 2C). Furthermore, when we analyzed the association between the transcription levels of active loci of NeoERVs and different classes of the identified tissue‐specifically transcribed NeoERV in Figure 2C, we found that most of them in the ERVL and ERVL‐MaLR classes, namely 1360 ERVL and 2384 ERVL‐MaLR elements among 4323 total elements.

### Correlations between the transcription levels of NeoERV active loci and host genes

**Figure 3:**
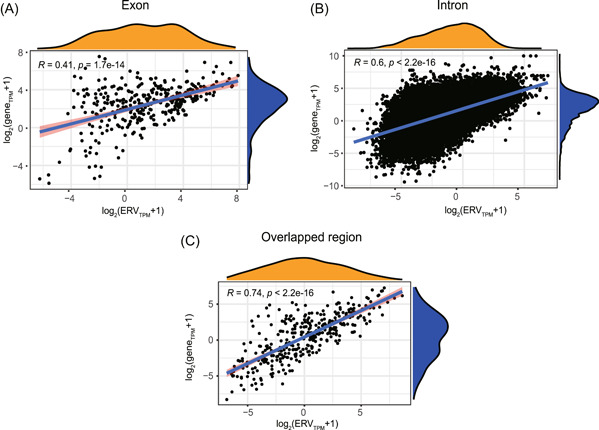
Correlations between the expression levels of active loci of NeoERV elements and host genes. (A) Active loci of NeoERVs located in exons. (B) Active loci of NeoERVs located in introns. (C) Active loci of NeoERVs located in overlapped regions.

In the human genome, LTRs are correlated with host gene expression^17^ and LTRs may be used as promoters or enhancers of host gene expression^18^. Correlations between the transcription levels of ERV elements and mammalian host genes are still largely unknown. In this study, we focused on the co‐transcription of active loci of NeoERV elements in the mink genome and calculated the correlation coefficients between the transcription levels of host genes and active loci of NeoERVs located in exons or introns or overlapped with host gene regions in lung, trachea, and amygdala tissues (Figure 3). Although the transcription of NeoERV elements in the intergenic regions might alter the transcription of their target host genes, these NeoERV elements were not considered in this study because of the challenge of confirming their target host genes. We found that the transcription levels of active loci of NeoERVs and related host genes were positively correlated across all three tissues examined. The correlation coefficients between the transcription levels of the active loci of NeoERVs and host genes were higher in lung tissue, especially for NeoERV elements in exon or intron regions. Furthermore, the transcription levels of active NeoERV loci that overlapped with host gene regions had the strongest positive correlations with host genes (*R* = 0.74, *p* < 2.2e−16). Overall, we found that the transcription levels of active loci of NeoERV elements were positively correlated with host gene transcription in all three types of tissues.

### Transcription of *env* genes of NeoERVs is higher in lung tissue

**Figure 4:**
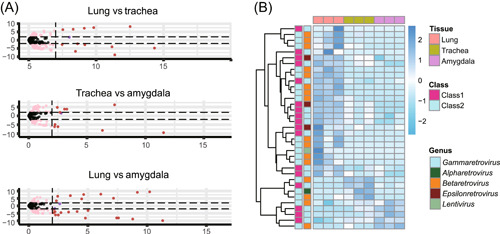
Expression levels of the *env* gene in three types of mink tissues. (A) Volcano plot showing differential *env* expression among three tissues. Differentially expressed *env* genes are highlighted in red. (B) Heatmap view of tissue‐specific expressed *env* genes and the corresponding retrovirus genera in the mink genome.

The *env* transcripts generated from ERV elements in mammals and avians are essential for protecting the hosts from exogenous retrovirus infection via receptor interference^19^. In this study, we investigated the *env* transcripts within NeoERV elements and *env* transcription levels in the three tissues. A total of 409 *env* transcripts were identified from 836 *env* sequences of NeoERV elements found to be active in the three tissues, which could potentially protect mink tissues from the seven genera of proviruses by interfering with receptors. We also analyzed the transcription levels of active *env* sequences in different tissues and showed that the transcription levels of 108 *env* genes were tissue‐specific in the three tissues (Figure 4A). We identified 35 active *env* sequences that were tissue‐specifically transcribed in the three tissues and belonged to five genera of retroviruses, *Gammaretrovirus*, *Alpharetrovirus*, *Betaretrovirus*, *Epsilonretrovirus*, and *Lentivirus* (Figure 4B). Most of the tissue‐specific *env* genes were transcribed in lung tissues (26 *env* genes), 4 were transcribed in trachea, and 5 were in amygdala. In addition, 11 tissue‐specific *env* genes encoded more than 70 peptides of the receptor‐binding surface domains, indicating that transcription of these *env* peptides may restrict retroviral infection^20^.

## DISCUSSION

Several bioinformatic tools can be used to identify ERVs in mammalian genomes, including RepeatMasker, RetroTector, and LTRfinders. We used RepeatMasker for the genome‐wide analysis of the mink genome because it has been widely used to identify ERV elements in mammals^21^, ^22^, ^23^, ^24^, ^25^, ^26^. ERVs coevolve with their host genomes and accumulate numerous mutations, including single‐nucleotide variations, insertions, and deletions. Most of the ERV elements are dispersed in their host genomes; therefore, only a small proportion of them are relatively complete provirus sequences. RepeatMasker uses the Repbase data set as a reference database because it contains all the annotated ERVs of mammalian genomes. Therefore, RepeatMasker can identify single LTRs that other software often fail to detect^27^. RepeatMasker is not suitable for proviral ERV sequence identification because the similarity search mode does not provide composition information of the ERV elements. Therefore, if the RepeatMasker is used for proviral ERV identification, add‐ons software is needed. We used RepeatMasker in this study because the focus was not on proviral ERV analysis.

ERV elements were long‐believed to be inactive until the discovery of ERV transcription in host cells, and ERV elements that produce transcripts should be considered active and could be involved in host gene expression^28^. In our study, we identified a total of 365,791 NeoERV loci in the mink genome of three tissues by genome‐wide scanning of ERV elements. However, the number of these loci is likely to change because the number of mammalian ERVs will increase or decrease with the development of the reference database. The whole‐genome transcriptomic data generated in this study were used to identify active NeoERV loci in three types of tissues, and these loci accounted for approximately one third of the total NeoERV loci. Clearly, by scanning the genome of other types of tissues, the numbers of NeoERV active loci are likely to increase because the number of identified tissue‐specific active loci would increase accordingly. The remaining ERV loci were the inactive loci, some of which may have been inactivated by epigenetic silencing and others could be permanently inactive because of high mutation levels. Among the active NeoERV loci identified in the three tissues, some tissue‐specific active loci were shared by two types of tissues and some were core active loci that were shared by all three tissues. The number of core active loci is expected to decrease as the number of investigated tissue types increases.

Comparative analysis of the differentially transcribed core active loci identified in the three tissues showed that approximately 87% of these loci had similar expression levels in all three tissues; hence, they were considered constant core active loci. The expression levels of the remaining core active loci increased in only one type of tissue, which were considered tissue‐specifically expressed core active loci. Loci that did not belong to either of these two categories showed increased transcription levels in two types of tissues. Therefore, the tissue‐specific expressed active loci consisted of two subsets: tissue‐specific expressed core active loci and tissue‐specific active loci.

Comparative analysis of transcription levels between NeoERVs and host genes indicated that the transcription levels of the active loci of NeoERV elements were positively correlated with those of host genes, especially when the NeoERVs were in intronic regions or overlapped gene regions. The active loci located in exons were also positively correlated with the host genes but at a lower level, indicating that these active loci may be co‐expressed with host genes. The NeoERV elements in intronic or overlapped regions may be used as alternative promoters to generate new alternative isoforms of the host gene, thus increasing the total transcription level of the host gene. Another important mechanism of active loci of NeoERV elements is the production of noncoding RNAs, such as HERV‐H in human embryonic stem cells^29^, which has been shown to regulate the expression of host genes. It is worth noting that the weaker positive correlation with host genes observed for the active loci in exons could be caused by alternative splicing under certain selective pressures.

The *env* gene encodes the envelope protein of ERVs. Envelope proteins are important antigens of ERVs, and the transcription of *env* genes in host genome could influence the immunity of the host toward possible infection by blocking virus receptors. We found that approximately 50% of all *env* loci (409/836) in the whole mink genome were active loci in all three types of tissues. The ratio of active loci to *env* loci was higher than that of total active NeoERV locus elements. A possible explanation for these differences is that the *env* genes have real functions in the mink immune system.

In summary, we identified NeoERV elements in the whole mink genome for the first time, and further identified active NeoERV loci by analyzing whole‐genome transcriptomic data. We found that more than one third of all active loci were tissue‐specifically transcribed. Furthermore, the comparative analysis showed there was a strong positive correlation between the transcription levels of active loci and host genes, especially for active loci located in overlapped gene regions of the mink genome. The in‐depth analysis of the transcription of *env* loci in the three selected tissues showed that the ratio of active loci was higher than that of total NeoERV loci, indicating that the *env* loci could have actual functions in mink immunity.

## MATERIALS AND METHODS

### Genomic sequence

The draft genome sequence and the annotated file of *Neogale vison* (GCA_020171115.1 ASM_NN_V1) downloaded from the National Center for Biotechnology were used to identify the NeoERV elements.

### Identification of mink ERVs

We identified NeoERV elements in the mink genome using RepeatMasker software (http://repeatmasker.org/RepeatMasker/), and the classes of NeoERV elements were annotated using the Repbase database^14^. To locate the NeoERV sequences in the mink genome, the GTF annotation file of the mink genome was used to identify intergenic, intragenic, and overlapped gene regions in the genome sequence. For intergenic regions, we used McSplicer^30^ to estimate the refined exon regions from multiple mink gene transcripts and then refined intronic regions based on the exon locations. The locations of the identified NeoERV elements were categorized at two levels: 1) all NeoERV elements that were located in intergenic, intragenic, or overlapped gene regions; and 2) NeoERV elements in intergenic regions were further located within exons, introns, or other regions; i.e., NeoERVs that have overlapped one or more exons.

### RNA sequencing (RNA‐seq)

Three six‐month‐old minks were used in our study. Tissue samples were collected from lung, trachea, and amygdala and frozen at −80°C for later use. Total RNA was isolated using a RNeasy mini kit (Qiagen) according to the manufacturer's protocol. High‐depth RNA‐seq sequencing was performed using a NovaSeq system (Illumina) to produce 150 bp paired‐end reads. A total of 70 Gb of reads were obtained for each tissue sample. The reads were trimmed using Trim Galore v0.6.4 (https://github.com/FelixKrueger/TrimGalore) for downstream analysis.

### RNA‐seq analysis

To investigate the expression levels of host genes and NeoERVs in different mink tissues, we aligned the trimmed reads to the mink genome using STAR v2.7 software^31^. For host genes, gene‐level read counts were obtained using the htseq‐count function in HTseq software^32^. The expression of the NeoERVs was analyzed using the aligned RNA‐seq data by adding the NeoERV locations from RepeatMasker, and the gene‐level read counts were obtained using the htseq‐count function in HTseq software. The read counts of host genes and NeoERV elements were converted to TPM values according to Wagner et al^33^. Finally, the differentially expressed NeoERVs were identified using the DESeq2^16^ with cutoffs of false discovery rate < 0.05 and fold change > 2.

### Identification of the *env* loci in NeoERV elements

We used the amino acid sequences corresponding to the *env* loci of 46 representative ERVs as queries to search the Gypsy Database (GyDB) of mobile genetic elements^34^. The *env* loci were searched in each NeoERV element using the tBLASTn^35^. When multiple *env* sequences were aligned to one location of a NeoERV element, the *env* gene with the longest hit was used to identify the genera of the *env* sequences of the NeoERV elements. Quantification of *env* gene expression levels and the corresponding differential expression analysis were conducted following the workflow for expression analysis of NeoERV elements.

### Statistical analysis and data visualization

All statistical analyses were performed with R statistical programming languages. PCA was conducted using the *prcomp* function in R, and visualization was performed using the *ggplot2* package. The volcano plot illustrating the differential expression levels of NeoERVs and *env* genes was obtained using the *EnhancedVolcano* package. A Venn diagram of the expression of active loci was generated using the *ggvenn* package. Heatmaps of differentially expressed *env* genes were generated using *pheatmap*.

## AUTHOR CONTRIBUTIONS

**Zheng Li**: Investigation (equal); writing—original draft (lead). **Qing Wang**: Investigation (equal). **Na Lv**: Data curation (equal). **Guojin Xu**: Data curation (equal); investigation (equal). **Xuemei Yang**: Data curation (equal). **Baoli Zhu**: Funding acquisition (lead); investigation (equal); project administration (lead); writing—review and editing (equal).

## ETHICS STATEMENT

All procedures in this study were conducted in accordance with the China Agricultural University Laboratory Animal Welfare and Animal Experiment Ethics Committee's (AW80203202‐1‐2) approved protocols.

## CONFLICT OF INTERESTS

The authors declare no conflict of interests.

## Supporting information

Supporting information.

## Data Availability

The raw RNA‐seq data that support the findings of this study are openly available in the NCBI SRA database, reference number PRJNA940981.
