# Supplementary material for: Genome‐wide identification of endogenous retrovirus elements and their active transcription in mink genome
Source: mLife. 2023 Jun 30;2(2):201–8. doi: 10.1002/mlf2.12074 (PMC10989824; doi:10.1002/mlf2.12074)
Supplement: Supplementary file 1 — Supporting information. [file MLF2-2-201-s001.docx]

Supplementary table 1: The distribution of active/inactive loci of NeoERV elements in 3 types of tissues.

| Tissues | Active loci | | | | Inactive loci | | | |
| --- | --- | --- | --- | --- | --- | --- | --- | --- |
|  | Intragenic | Overlap region | Intergenic | Other | Intragenic | Overlap  region | Intergenic | Other |
| Lung | 47941 | 282 | 65654 | 631 | 55784 | 257 | 194146 | 1096 |
| Trachea | 48014 | 285 | 70304 | 604 | 55711 | 254 | 189496 | 1123 |
| Amygdala | 38440 | 230 | 47604 | 491 | 65285 | 309 | 212196 | 1236 |
| All | 161205 | | | | 204586 | | | |

Other represent the location of NeoERV loci has complex relationships between more than one genes. All represents the sum of active/inactive loci in 3 types of tissues after removing redundancy.
